# Concentration gradients of monoamines, their precursors and metabolites in serial lumbar cerebrospinal fluid of neurologically healthy patients determined with a novel LC–MS/MS technique

**DOI:** 10.1186/s12987-023-00413-8

**Published:** 2023-02-13

**Authors:** Celien Tigchelaar, Willemien D. Muller, Sawal D. Atmosoerodjo, Klaas J. Wardenaar, Ido P. Kema, Anthony R. Absalom, Martijn van Faassen

**Affiliations:** 1 Department of Anaesthesiology, University Medical Center Groningen, University of Groningen, Hanzeplein 1, 9713 GZ Groningen, The Netherlands; 2 Department of Psychiatry, University Medical Center Groningen, University of Groningen, Groningen, The Netherlands; 3 Department of Laboratory Medicine, University Medical Center Groningen, University of Groningen, Groningen, The Netherlands

**Keywords:** Biogenic amines, Cerebrospinal fluid (CSF), Concentration gradients, LC–MS/MS

## Abstract

**Background:**

Potential biomarkers for neuropsychiatric disorders are cerebrospinal fluid (CSF) monoamines and their corresponding precursors and metabolites. During CSF sampling, CSF flows towards the lumbar sampling site from more cranial regions. To compare the results of studies in which different CSF volumes were acquired, it is important to know if ventricular-lumbar concentration gradients exist. This has only been addressed for a few biogenic amines, and almost exclusively in neurologically unwell patients due to the burden of a lumbar puncture (necessary to obtain CSF). The aim of our study was to determine if concentration gradients exist for routinely measured CSF constituents and biogenic amines in neurologically healthy patients. We applied a novel ultrasensitive liquid chromatography mass spectrometry (LC–MS/MS) method for the simultaneous quantification of multiple monoamines, precursors and metabolites in CSF and plasma.

**Methods:**

CSF and blood samples were collected from twenty neurologically healthy patients undergoing spinal anaesthesia. Ten mL of lumbar CSF was collected in five consecutive two mL fractions. We determined leucocyte and erythrocyte counts, glucose, albumin and protein concentrations and quantified monoamines, precursors and metabolites on each of the fractions using LC–MS/MS.

**Results:**

In twenty patients (60% male; median age: 46 years), dopamine, DOPAC, 3-MT, HVA, noradrenaline, normetanephrine and 5-HIAA concentrations increased from the first to the last CSF fraction (all p < 0.001). CSF adrenaline concentrations were below the detection limit, whereas serotonin measurements were regarded as unreliable. Albumin and total protein levels decreased significantly across CSF fractions.

**Conclusions:**

A ventricular-lumbar CSF concentration gradient existed for most of the investigated analytes. This is a novel finding for dopamine, noradrenaline, 3-MT and normetanephrine. These results contribute to the understanding of the neurobiology and underline the importance of standardized procedures for CSF handling to allow comparisons between studies.

**Supplementary Information:**

The online version contains supplementary material available at 10.1186/s12987-023-00413-8.

## Background

In healthy adults, approximately 500 mL of cerebrospinal fluid (CSF) is produced daily by the choroid plexuses, circulates to the subarachnoid space [1] and is absorbed by the arachnoid villi [2]. For many decades, CSF leucocyte counts, and assays of glucose, albumin and protein have been routinely used for differential diagnostics in neurology [3]. The CSF/blood albumin quotient (Qalb) has been used as indicator of blood-CSF-barrier (BCB) permeability [4], and has been found informative for a variety of inflammatory conditions [5]. More recently it has become apparent that analysis of CSF composition can help illuminate the neurobiology of central nervous system (CNS) disorders and identify novel biomarkers [6, 7].

Potential biomarkers of interest are monoamines and their corresponding precursors and metabolites [8, 9]. Major monoaminergic systems include the serotonergic, dopaminergic and (nor)adrenergic systems. Serotonin (5-HT) and its main metabolite 5-hydroxyindoleacetic acid (5-HIAA) are implicated in the etiology of depression [10–12] and suicidality [13, 14]. Alterations in levels of dopamine (DA), its precursor levodopa (L-DOPA) and metabolite 3,4-dihydroxyphenylacetic acid (DOPAC), are associated with Parkinson’s disease (PD) [15]. Alterations in the concentration of homovanillic acid (HVA), another dopamine metabolite, are associated with mitochondrial diseases [16], and a broad variety of psychiatric problems including depression [10, 12], bipolar disorder [17] and suicidality [14]. (Nor)adrenaline concentrations are considered as marker for neuropsychiatric disorders, with recent studies showing reduced concentrations in patients with cognitive decline [18]. A metabolite of noradrenaline (NA), 3-methoxy-4-hydroxyphenylglycol (MOPEG), has been associated with PD [15] and seems promising in differentiating between Alzheimer's disease and other types of dementia [19]. In several inherited metabolic diseases biogenic amine synthetic pathways have been shown to be affected [20].

Despite increasing evidence of disturbances in monoamine signaling in the pathophysiology of neuropsychiatric disorders [8, 9], research results should be interpreted carefully. When combining or comparing the results of biomarker studies, differences in CSF collection and aliquoting strategies could potentially bias conclusions if the concentration of the molecule is not uniform throughout the CSF [21, 22]. Whereas initially acquired CSF will mostly be from the lumbar site, later samples include CSF from more rostral (cranial) regions. A rostrocaudal concentration gradient (RCG) is thought to develop as a consequence of molecular-size-dependent and concentration driven diffusion throughout the CNS [23]. Blood derived proteins have net flux from blood into CSF throughout the CNS. CSF flows in a cranio-caudal direction, and as it does so, concentrations of blood-derived proteins gradually increase [23]. Conversely, concentrations of brain-derived proteins tend to be highest in ventricular CSF and lower in the lumbar CSF [21, 23].

Previous studies initially focused on blood-derived proteins and demonstrated a marked decrease in albumin and Immunoglobulin G (IgG) concentrations from the first to the last lumbar CSF fraction [24, 25]. Subsequently, the focus shifted to brain-derived proteins functioning as neurological biomarkers, such as amyloid-β (Aβ) [26, 27] and Tau [23, 27]. Literature regarding biogenic amines is still scarce and mostly concerns the metabolites HVA, 5-HIAA and MOPEG. In summary, these studies have found pronounced RCG’s for HVA and 5-HIAA, with linearly increasing concentrations across subsequent CSF fractions [28–33]. For MOPEG, evidence is inconsistent [28, 29, 31–33] and most studies have found no significant gradient [29, 31, 33]. There are as yet no published analyses of concentration gradients for other relevant metabolites such as 3-o-methyldopa (3-OMD), 3-methoxytyramine (3-MT), normetanephrine (NMN) or metanephrine (MN). Only one study investigated gradients in monoamine neurotransmitters and demonstrated a significantly elevated concentration in the second CSF fraction compared to the first fraction for serotonin, dopamine, and DOPAC, but not for noradrenaline [29].

The literature in this field is not only scarce, it also almost exclusively reports data from patients with (suspected) neurological disease [25, 26, 28, 34] due to the burden of a lumbar puncture necessary to obtain CSF. Furthermore, most studies were conducted more than twenty years ago and only involved analysis of one or a few amines [28, 31, 33]. We have recently developed a state of the art method using liquid chromatography with tandem mass spectrometry (LC–MS/MS) [35, 36] that requires only small volumes of plasma or CSF to perform sensitive and simultaneous quantification of all relevant biogenic amines. The objective of our study was to use this technique to determine if a concentration gradient exists for CSF monoamines, their precursors and metabolites in neurologically healthy patients. Our secondary objective was to verify if a concentration gradient exists for frequently used routine CSF analyses.

## Methods

### Study design

This study is a sub-study of the Anaesthetic Biobank of Cerebrospinal Fluid (ABC) of the University Medical Center Groningen (UMCG), The Netherlands. The purpose of the ABC is to collect and store CSF of relatively (neurologically) healthy adult patients for future neuroscientific research. The ABC was approved by the Medical Ethical Committee of the UMCG (registration number 2016-174). Details of the main study design have been described in detail [6].

### Study population

All patients 18 years of age and older scheduled for elective surgery under spinal anaesthesia were approached for participation in the ABC study, except for patients scheduled for caesarean section. After informed consent was obtained, all patients were also screened for eligibility for this sub-study. Patients with relevant neurological, psychiatric, metabolic (e.g. diabetes mellitus) or inflammatory disorders, or relevant medication use (e.g. antidepressants, antipsychotics, immunosuppressive drugs, dopamine antagonists) were excluded. Additional exclusion criteria were: Body Mass Index (BMI) ≥ 30 kg/m^2^, pregnancy, excessive smoking (more than five cigarettes daily), alcohol abuse (men: more than two units per day; women: more than one unit per day), and recreational drug use. Patients were excluded if the procedure was performed in lateral position, blood was visible in the CSF, less than 10 mL CSF was collected or CSF aspiration time exceeded 4 min. Those patients excluded for this study still participated in the ABC study.

### CSF and blood collection and handling

Spinal anaesthesia was conducted using a standard protocol [6]. Prior to local anesthetic injection, 10 mL CSF was aspirated into five 2 mL syringes. The first 1 mL of each consecutive 2 mL fraction was immediately used for routine analyses. The remaining 1 mL of each fraction was transferred into a polypropylene tube wrapped in aluminium foil and directly put on ice for transport to the laboratory where it was centrifuged (1000 ×*g*, 10 min, 4 ℃) and stored in two aliquots of 500 µL at − 80ºC. For all five fractions, glutathione was added to one of the two aliquots for stabilization.

Blood (20.5 mL) was collected during intravenous cannulation prior to surgery, of which 10.5 mL was sent immediately for routine analyses. The remaining 10 mL was collected in a 10 mL EDTA tube, directly put on ice for transport to the laboratory where it was centrifuged (2000 ×*g*, 10 min, 4 ℃). The cell-free plasma was stored in ten aliquots of 500 µL at – 80 ℃. Glutathione and Dithiothreitol (DTT) were added to two aliquots for stabilization.

### Laboratory analysis

Monoamines, precursors and metabolites assayed included L-DOPA, 3-OMD, dopamine, DOPAC, 3-MT, HVA, noradrenaline, normetanephrine, MOPEG, adrenaline, metanephrine, 5-HIAA and serotonin. Concentrations of all analytes were analysed using online solid-phase extraction in combination with LC–MS/MS as previously described [35, 36]. In short: 50 µL plasma or CSF was pipetted in a 96-well plate mixed with 50 μL of internal standard working solution, 250 μL of 0.5 mol/L dipotassium phosphate, and 4 mmol/L K2EDTA, pH 8.5. Subsequently, 50 μL of 25% (v/v) propionic anhydride in acetonitrile was added, and the plate was vortex mixed for 15 min. Water was added to all wells to a total volume of 1.0 mL. The plate was vortex mixed and centrifuged for 15 min at 1500 g. Then, 50 μL of each calibrator and sample was injected onto the online solid-phase extraction (SPE) LC–MS/MS system. Intraassay imprecision was < 7.1% at three different concentrations for L-DOPA, 3-OMD, catecholamines and metanephrines (n = 20). Limit of quantification (LOQ) was 1, 1, 0.01, 0.01, 0.03, 0.01, 0.05, 0.04 nmol/L for L-DOPA, 3-OMD, DA, noradrenaline, adrenaline, 3-MT, normetanephrine, metanephrine, respectively. For the indoles, intraassay imprecision was < 6.1% at three different concentrations (n = 20). LOQ was 0.01 nmol/L for serotonin, and 15 nmol/L for 5-HIAA. DOPAC, HVA and MOPEG were analysed essentially as described by Ford et al. [37]. The same online SPE system in combination with LC–MS/MS was used as described above. Intraassay imprecision was < 4% for at three different concentrations (n = 10) for DOPAC, HVA, and MOPEG. LOQ was 0.06, 0.05, and 0.13 nmol/L for DOPAC, HVA, and MOPEG, respectively. All samples were analysed in the same analytical run to minimize contribution of analytical variation, with only one freeze thaw cycle. As the serotonin concentration in CSF is much lower than in platelet-rich plasma, the analysis for serotonin had to be repeated with optimal mass spectrometer settings. For the serotonin analysis, most of the samples underwent two freeze–thaw cycles and for a small part of the samples, three freeze–thaw cycles were necessary due to an error in the sample preparation step.

Routine CSF and plasma analyses included glucose, leucocyte count, albumin and total protein concentrations. CSF erythrocyte counts were determined, to assess for blood contamination (due to the spinal puncture) [22].

### Data collection

Data concerning patient demographics, medical history, medication use and the American Society of Anaesthesiologists (ASA) score [38] were obtained from patient records. If logistically possible, the Montreal Cognitive Assessment (MoCA) was performed preoperatively. Details of the lumbar puncture and CSF collection were recorded.

### Sample size

Based on the sample size of 20 and five fractions (repeated measures) we calculated the statistical power to detect a medium within-subject effect for fraction (i.e. gradient; η^2^ = 0.06; [39]) in a RM-ANOVA, using G*Power [40]. With an alpha of 0.05, and expected (test–retest) correlation among repeated measurements at 0.7, the expected power was 0.96. In addition, the minimum effect that could be tested with a power of 0.8 was η^2^ = 0.04 (4% of total variance accounted for by fraction).

### Statistical analysis

Analyses were performed using SPSS statistics software (version 26; IBM Corp., Armonk, New York, Unites Stated of America). Continuous data are presented as mean ± standard deviation or as median [inter quartile range (IQR)] when appropriate, discrete data by category frequencies (%). For every normally distributed laboratory measurement, a possible linear RCG was investigated by running a repeated measures ANOVA (laboratory measurement as dependent variable and fraction as independent within-subject factor). Analyses were checked for assumption violations. To test the within-subject effect, the Greenhouse–Geisser correction was used in case the assumption of sphericity was violated (according to Mauchly’s test). To assess the within-subject differences between the first and each of the subsequent fractions, post hoc pairwise comparisons were performed. P-values were adjusted for multiple testing using the Bonferroni correction. For non-normally distributed outcomes, a Friedman test with post hoc Wilcoxon signed-rank tests was used to investigate the presence of within-subject differences between fractions. Pearson/Spearman correlation coefficients were calculated to investigate the relationships between blood and CSF fractions. Qalb was calculated by dividing the concentration of albumin in CSF by the concentration of albumin in blood, times one thousand.

## Results

Between June 2019 and February 2020, 79 patients were enrolled for the ABC and screened for eligibility for this sub-study. Of them, 59 were considered ineligible for one or more of the following reasons: BMI > 30 kg/m^2^ (20 patients), relevant medical history or medication use (38 patients), excessive smoking, alcohol or drug use (9 patients). Additionally, five patients were excluded during the spinal procedure, because of failure to aspirate 10 mL CSF within 4 min. Patient characteristics of the 20 patients included in this study are shown in Table 1. Mean age was 46 years (range 21–77) and 60% were male. Fourteen patients were classified as ASA physical status I. All patients scored in the normal range of the MoCA (≥ 26). The lumbar punctures were mostly performed at level L3–L4 and aspiration of 10 ml CSF required on average 2 min. CSF samples were collected between 8 am and 2 pm.

**Table 1:** Patient characteristics

N	20
Baseline characteristics	
Age (years)	46 ± 17.6; range 21–77
Sex, male	12 (60%)
Ethnicity	
Caucasian	19 (95%)
African	1 (5%)
Weight (kg)	79 ± 12
Height (cm)	176 [170–184]
BMI (kg/m^2^)	25 ± 3; range 19–29
ASA classification	
ASA I	14 (70%)
ASA II	6 (30%)
Lifestyle factors	
Current smoking	1 (5%)
Past smoking	9 (45%)
Alcohol use	17 (85%)
No medication use	9
MoCA score	27 [26–29]
Surgical characteristics	
Type of surgery	
Urological	1 (5%)
Orthopaedic	17 (85%)
Surgical	2 (10%)
Spinal technique characteristics	
Puncture level	
L1–L2	1 (5%)
L2–L3	2 (10%)
L3–L4	13 (65%)
L4–L5	4 (20%)
CSF aspiration time (minutes)	2 [2, 3]; range 1–4

Data are presented as n (%), mean ± SD or median [IQR]

*BMI* Body Mass Index, *ASA* American Society of Anaesthesiologists, *MoCA* Montreal Cognitive Assessment, *CSF* Cerebrospinal fluid

### Biogenic amine concentrations and gradients

Metanephrine and adrenaline concentrations were quantifiable in plasma, but were below the quantification limits in CSF (0.04 nmol/L and 0.03 nmol/L respectively).

**Fig. 1:**
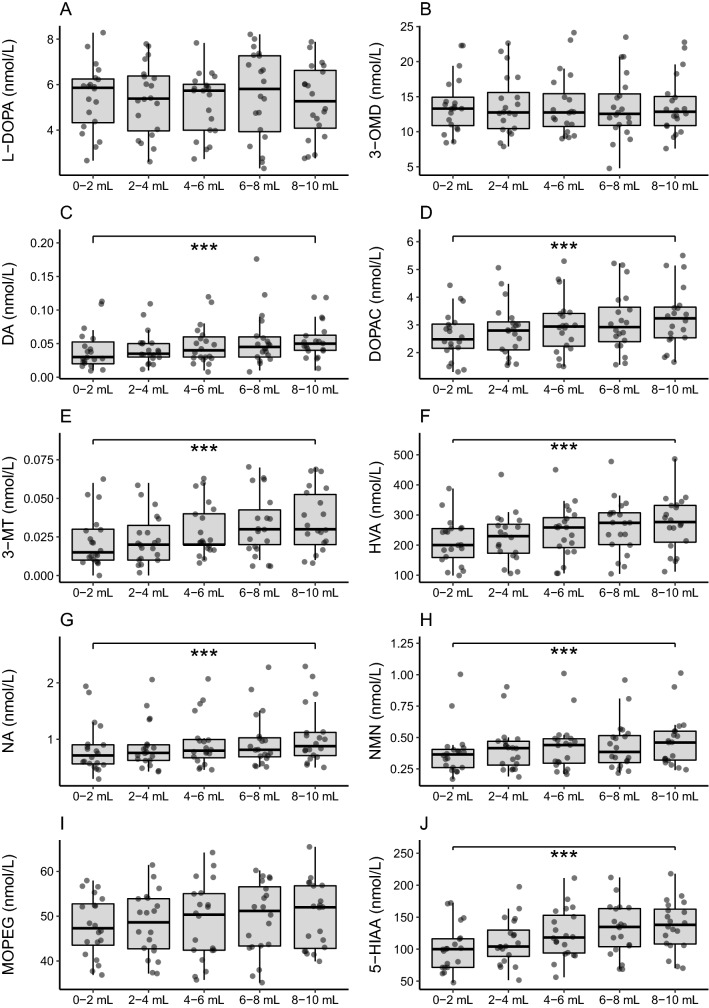
Concentrations of monoamines, their precursors and metabolites in five consecutive CSF fractions. Concentrations of **A** L-DOPA (levodopa), **B** 3-OMD (3-o-methyldopa), **C** DA (dopamine), **D** DOPAC (3,4-Dihydroxyphenylacetic acid), **E** 3-MT (3-methoxytyramine), **F** HVA (homovanillic acid), **G** NA (noradrenalin), **H** NMN (normetanephrine), **I** MOPEG (3-Methoxy-4-hydroxyphenylglycol) and **J** 5-HIAA (5-hydroxyindoleacetic acid) for the five consecutive fractions of CSF: 1: 0–2 mL, 2: 2–4 mL, 3: 4–6 mL, 4: 6–8 mL, 5: 8–10 mL. For all analytes, the bold line shows the median concentration and the grey box the IQR. Concentration gradient for analytes significant at 0.05 level (*), 0.01 level (**) or 0.001 level (***)

Concentrations of dopamine, DOPAC, 3-MT, HVA, noradrenaline, normetanephrine and 5-HIAA increased significantly across CSF fractions (all p < 0.001) (Table 2 and Fig. 1). A substantially higher concentration in the fifth compared to the first fraction was found for dopamine (+ 67%:), DOPAC (+ 26%), 3-MT (+ 50%), HVA (+ 29%), noradrenaline (+ 9%), normetanephrine (+ 24%) and 5-HIAA (+ 35%). Although a similar pattern was observed for CSF MOPEG, these differences across CSF fractions did not reach statistical significance (p = 0.054). There were also no significant concentration gradients across fractions for L-DOPA and 3-OMD. For the concentrations of most molecules there was a large degree of inter-individual variability, but a much smaller intra-individual variability, with clear concentration trends across fractions in several cases (Additional file 1: Fig. S1).

**Table 2:** Concentrations of analytes in plasma and five consecutive CSF fractions

N	20	20	20	20	20	20	*p-value*	*F (df, error df)*	*χ*^*2*^* (df)*
	Plasma	CSF 0–2 mL	CSF 2–4 mL	CSF 4–6 mL	CSF 6–8 mL	CSF 8–10 mL			
Monoamines, precursors and metabolites									
L-DOPA (nmol/L)	17.0 [14.1–31.4]	5.44 (4.75–6.13)	5.37 (4.64–6.10)	5.20 (4.55–5.84)	5.55 (4.65–6.46)	5.26 (4.51–6.01)	0.426	0.93 (4, 76)	
3-OMD (nmol/L)	96.4 (84.5–108.3)	13.3 [10.6–16.2]	12.8 [10.4–17.0]	12.8 [10.3–16.5]	12.6 [10.7–16.0]	12.9 [10.8–15.3]	0.981		0.41 (4)
DA (nmol/L)	0.09 (0.08–0.11)	0.031 [0.020–0.054]	0.035 [0.025–0.052]	0.040 [0.026–0.059]	0.047 [0.030–0.056]	0.050 [0.037–0.063]	< 0.001***		42.12 (4)
DOPAC (nmol/L)	9.61 (7.24–11.99)	2.60 (2.20–3.00)	2.80 2.35–3.24)	2.96 (2.49–3.44)	3.11 (2.60–3.62)	3.28 (2.77–3.79)	< 0.001***	18.5 (1.85, 35.10)	
3-MT (nmol/L)	0.02 (0.06–0.05)	0.016 [0.011–0.029]	0.018 [0.012–0.035]	0.022 [0.016–0.039]	0.028 [0.017–0.050]	0.031 [0.022–0.056]	< 0.001***		38.3 (4)
HVA (nmol/L)	69.6 (58.8–80.3)	211 (175–246)	225 (188–262)	245 (205–285)	260 (218–302)	272 (230–314)	< 0.001***	28.1 (1.60, 30.4)	
NA (nmol/L)	2.54 (0.82–1.30)	0.81 [0.58–0.91]	0.76 [0.62–0.90]	0.80 [0.64–1.01]	0.81 [0.67–1.04]	0.88 [0.70–1.13]	< 0.001***		39.8 (4)
NMN (nmol/L)	0.40 [0.35–0.47]	0.37 [0.26–0.41]	0.42 [0.26–0.47]	0.44 [0.28–0.49]	0.38 [0.28–0.53]	0.46 [0.32–0.55]	< 0.001***		42.937 (4)
MOPEG (nmol/L)	22.2 (19.7–24.6)	47.7 (44.6–50.9)	48.2 (44.8–51.5)	48.8 (44.8–52.8)	49.5 (45.8–53.3)	50.1 46.7–53.5)	0.054	2.44 (4, 76)	
ADR (nmol/L)	0.23 [0.15–0.43]	ND	ND	ND	ND	ND			
MN (nmol/L)	0.23 (0.18–0.28)	ND	ND	ND	ND	ND			
5-HIAA (nmol/L)	50.2 (44.3–56.1)	101.1 (84.4–117.8)	111.9 (95.2–128.5)	124.4 (106.4–142.3)	130.3 (111.7–149.0)	136.7 (118.5–154.8)	< 0.001***	24.0 (1.57, 29.91)	
Routine laboratory analysis									
Albumin (g/L)	45.3 (43.3–47.2)	0.21 (0.18–0.25)	0.20 (0.17–0.24)	0.20 (0.17–0.20)	0.19 (0.15–0.22)	0.19 (0.15–0.22)	< 0.001***	16.936 (2.003, 38.054)	
Qalb (× 10^–3^)	–	4.73 (3.97–5.50)	4.54 (3.76–5.32)	4.42 (3.68–5.16)	4.13 (3.41–4.86)	4.15 (3.40–4.89)			
Total protein (g/L)	73.3 (70.6–75.9)	0.34 (0.30–0.39)	0.33 (0.29–0.38)	0.33 (0.28–0.37)	0.31 (0.27–0.36)	0.31 (0.26–0.36)	< 0.001***	11.393 (1.816, 34.509)	
Glucose (mmol/L)	5.25 (4.96–5.54)	3.31 (3.18–3.43)	3.34 (3.21–3.47)	3.33 (3.20–3.45)	3.34 (3.20–3.47)	3.33 (3.20–3.46)	0.047*	2.536 (4, 76)	
Erythrocyte count (× 10^6^/L)	–	200 [100–300]	150 [100–275]	100 [100–200]	100 [100–200]	100 [100–200]	0.083		8.230 (4)
Leucocyte count (× 10^6^/L)	5.95 (5.49–6.93)	1.00 [1.00–2.75]	1.00 [1.00–1.75]	1.00 [1.00–1.75]	1.00 [0.25–2.00]	1.00 [0.00–1.00]	0.001**		19.551 (4)

Data are presented as mean (95% CI) or median [IQR]. Test (repeated-measures ANOVA or Friedman’s test) is significant at 0.05 level (*), 0.01 level (**) or 0.001 level (***)

*L-DOPA* levodopa, *3-OMD* 3-o-methyldopa, *DA* dopamine, *DOPAC* 3,4-Dihydroxyphenylacetic acid, *3-MT* 3-methoxytyramine, *HVA* homovanillic acid, *NA* noradrenalin, *NMN* normetanephrine, *ADR* adrenalin, *MN* metanephrine, *MOPEG* 3-Methoxy-4-hydroxyphenylglycol, *5-HIAA* 5-hydroxyindoleacetic acid, *Qalb* ratio of CSF to plasma albumin concentration, *ND* non-detectable, *df* degrees of freedom

Although serotonin concentrations were detectable, we do not consider them sufficiently reliable for publication and do not report them. Samples from this study underwent a maximum of three freeze thaw cycles which resulted in degradation of serotonin. The serotonin concentrations that were measured were lower than published values [41] and were 5—tenfold lower than concentrations measured in a larger sample of the ABC biobank study (data not shown here). The samples from the ABC biobank study were analysed after only one thaw cycle.

CSF 3-OMD concentrations were strongly correlated with plasma concentrations (all CSF fractions p < 0.001) (Additional file 2: Table S1). There was no significant correlation between plasma and CSF concentrations for the remaining molecules (p = 0.051–0.992), except for MOPEG levels of the second CSF fraction (p = 0.012).

### Routine assays

**Fig. 2:**
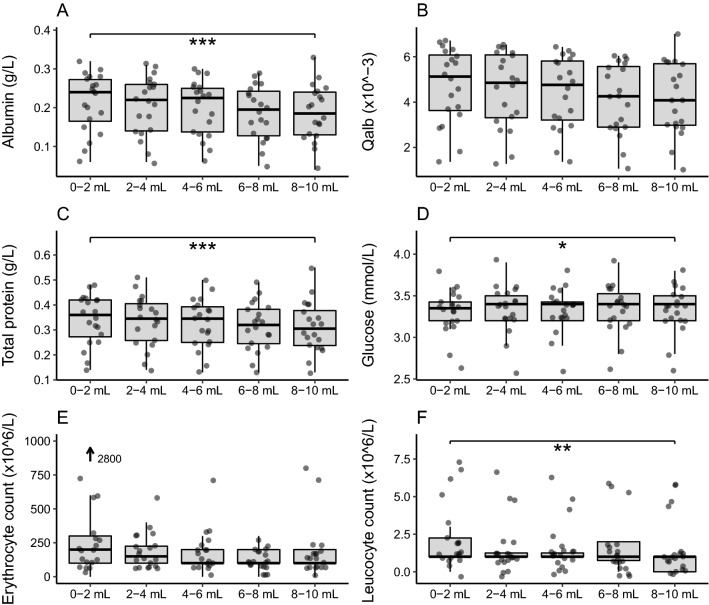
Concentrations of routine laboratory analyses in five consecutive CSF fractions. Concentrations of **A** albumin, **B** Qalb, **C** total protein, **D** glucose, **E** erythrocyte count and **F** leukocyte count for the five consecutive fractions of CSF: 1: 0–2 mL, 2: 2–4 mL, 3: 4–6 mL, 4: 6–8 mL, 5: 8–10 mL. For all analytes, the bold line shows the median concentration and the grey box the IQR. Concentration gradient for analytes significant at 0.05 level (*), 0.01 level (**) or 0.001 level (***). *Qalb* ratio of CSF to plasma albumin concentration

The results of routine assays are summarised in Table 2 and Fig. 2. Albumin concentrations decreased significantly from 0.21 g/L in the first 2 mL to 0.19 g/L in the last 2 mL of CSF (p < 0.001). Total protein concentrations showed a similar decrease, from 0.34 g/L in the first CSF fraction to 0.31 g/L in the last fraction (p < 0.001). For glucose concentration, erythrocyte and leucocyte count, no significant effect of CSF fraction was found. Erythrocyte count exceeded 500/μL in eight samples (8% of total), of which four samples of the first fraction, two of the fifth fraction and one sample of the second and third fraction. There was a moderate, positive correlation between plasma and CSF glucose concentrations (p = 0.002–0.004 for all five CSF fractions; Additional file 2: Table S1).

## Discussion

Concentrations of a panel of biogenic amines were measured simultaneously with a high sensitive LC–MS/MS method in five consecutive 2 mL lumbar CSF fractions in a relatively neurological healthy surgical population. We found novel evidence of concentration gradients for dopamine, noradrenaline, 3-MT and NMN, and confirmed prior findings of gradients for DOPAC, HVA, and 5-HIAA concentrations. For these molecules concentrations increased significantly from the first to the last fraction, suggesting a negative concentration gradient in a cranio-caudal direction. In contrast, albumin and total protein concentrations decreased significantly across the subsequent CSF fractions. For MOPEG, L-DOPA, 3-OMD, glucose, erythrocyte and leucocyte count, no clear gradient was observed. CSF concentrations of adrenaline, metanephrine and serotonin in CSF were not (reliably) measurable.

Our results are consistent with the theoretical framework [23] and reflect earlier observations, but the strength of evidence depends on the specific analyte. Regarding the serotonergic system, we confirmed a RCG for 5-HIAA. This finding is consistent with multiple studies with neurological patients [30, 31, 33], as well as with studies which only included healthy volunteers [29, 32].

For the dopaminergic system, we found similar results to those of Eklundt et al. who showed an higher level of dopamine and DOPAC in the second CSF fraction compared to the first fraction in fourteen male volunteers [29]. As dopamine is mainly produced in the CNS and is not able to cross the BCB, and DOPAC and 3-MT are metabolites of dopamine, the ventricular-lumbar gradient is as expected for these brain-derived molecules. Accordingly, L-DOPA crosses the BCB barrier, so it is not surprising that L-DOPA and its metabolite 3-OMD showed no gradient. We confirmed a concentration gradient for HVA, which is consistent with prior research [29–33].

Regarding the adrenergic system, our results showed a modest RCG for noradrenaline and normetanephrine. Eklundt et al. on the other hand found no gradient for noradrenaline (and did not study normetanephrine) [29]. Noradrenaline is an important neurotransmitter of the CNS and is present throughout the brain, but could also be derived from the spinal cord itself [42, 43]. Noradrenaline is also a major neurotransmitter of the peripheral sympathetic nervous system [43]. General consensus is that catecholamines are unable to cross the BCB [42, 44]. As our subjects were relatively healthy patients with a Qalb in the normal range, and the correlation between plasma and CSF noradrenaline was not significant, it is likely that noradrenaline in the CSF does not originate from the plasma. Our study supports previous work showing that CSF noradrenaline originates in the brain [23]. For MOPEG, although not significant, an increase in concentration across fractions was visible. One study reported a modest concentration gradient for MOPEG [28] and one study found a gradient in volunteers [32], but most studies concluded no gradient exist for MOPEG [29, 31, 33]. An explanation for a slight gradient might be that MOPEG can cross the BCB easily, thereby eliminating a concentration gradient [28, 45], which is supported by our finding of a moderate correlation between plasma and CSF MOPEG.

For our secondary objective, an identical concentration gradient was found as demonstrated in earlier studies for albumin and Qalb [24, 25, 34]. For total protein, most studies have investigated only one specific protein such as IgG, Aβ [26, 27] or Tau [27], and results are often contradictory [34, 46]. Nevertheless, as the main fraction of proteins in the normal CSF originates from blood (approximately 80%), and albumin constitutes up to 80% of total protein [23], a concentration pattern similar to albumin is therefore likely for total protein in healthy individuals, as this study confirms. These results for albumin and total protein are as expected, since these molecules are blood derived proteins. The transfer of proteins from blood to CSF happens by passive diffusion alongside the local concentration gradient, which is depending on the molecular size (small molecules have a higher penetration depth and therefore concentrations in CSF will increase). As CSF flows in a cranio-caudal direction, concentrations increase from cranial to caudal [23].

For the remaining routine analyses, no clear gradient effect was found. Presence of erythrocytes in CSF can indicate a haemorrhage, but is more commonly (~ 17%) the result of a ‘bloody tap’ [22]. As we mostly investigated brain-derived proteins, and median erythrocyte counts were < 500/μL for all fractions, and the individual samples with erythrocyte counts > 500/μL were scattered over the different patients and fractions, this probably did not influence the results. Whereas many studies do not use the first 1–2 mL of CSF to avoid possible blood contamination [47], our finding that different fractions were contaminated suggests that this is not a successful strategy. The lack of a gradient in leukocyte count was consistent with expectations, as very few leukocytes are usually present in CSF of healthy individuals. We also found no gradient for CSF glucose. Although evidence on gradients is lacking, this was also an expected finding, given that glucose is actively transported across the BCB [2], which is also reflected by the correlation we found between plasma and CSF glucose.

As has been mentioned previously, our subjects were relatively healthy patients. The extent of inter-individual variability in concentrations for all fractions was remarkable. In some cases (e.g. NMN) up to tenfold variability was seen. Despite this inter-individual variability, within-patient variability (i.e. differences across fractions) was only modest.

This study has several strengths. Although metabolite concentrations are a reflection of monoamine turnover, the concentrations of the primary monoamines are preferred due to their functional status [28]. Ours is one of the few studies regarding gradients which has determined concentrations of dopamine and noradrenaline. Second, we selected relatively healthy neurological patients, and comparison data from healthy controls is necessary to help interpret the findings of CSF biomarker studies. Third, this study demonstrated that levels of an entire panel of catecholamines and metanephrines can be measured more precisely today in a small volume of CSF with LC–MS/MS, which has not been done before. Lastly, CSF was obtained using standardized collection methods, and exclusion criteria were handled to attain ‘clean’ CSF samples.

Some study limitations deserve mention. First, only ten mL of lumbar CSF was collected, which is similar to the total volume of CSF below the conus medularis [48]. This limits the confidence with which conclusions can be made about cranio-caudal concentration gradients and ventricular concentrations. Ventricular CSF has rarely been studied since it is difficult to obtain. A few previous studies have investigated differences between the composition of ventricular and lumbar CSF, mostly in patients with an external ventricular drain or ventriculoperitoneal shunt for hydrocephalus. These studies found similar results as our study regarding the ventricular-lumbar concentration gradient of routine analyses [49, 50]. This study also found evidence for rostrocaudal gradients of monoamines similar to one previous study including ventricular CSF [51]. As most of our results also match with results of previous studies and our results are consistent with the theoretical framework [23], we believe that our results can be extrapolated to estimate ventricular concentrations. Moreover, most previous studies collected lumbar CSF, so this does not limit the comparison with literature. Also, during a lumbar puncture for diagnostic purposes, usually between five to fifteen mL CSF is obtained, meaning that results of this study could also have clinical implications. Second, our participants cannot be interpreted as completely healthy volunteers as our patients had surgery, even though we did apply strict exclusion criteria and our data supports this (e.g. normal range MoCA scores). Third, several causes of variation in monoamine concentration in CSF have been described with varying strength of evidence [52], for instance for age [53, 54], sex [32, 53], height [53, 55], weight [55] and CSF aspiration time [30, 56]. Our study did select patients on the basis of some of these factors (e.g., aspiration time < 4 min) but we could not take into consideration all possible confounding factors of analyte concentrations.

This study implies that concentrations of numerous CSF monoamine, precursors and metabolites, and routine analytes, will vary depending on the volume of CSF that is collected. If a gradient exists, standardized procedures for CSF collection and handling are essential to allow comparisons between studies and investigators should acquire CSF in discrete fractions and carefully report their CSF acquisition methodology [22]. To ensure that results from combining samples of multiple studies are not influenced by preanalytical factors, we suggest future studies to follow the consensus protocol from Teunissen et al. for the standardization of CSF biobanking [22]. Dependent on the feasibility of certain study methods in clinical practice, a future study can decide to deviate from this protocol (as for instance we collected 10 mL CSF instead of the suggested > 10 mL). Also, possible confounding factors affecting biogenic amine concentrations could be investigated in a larger study and reliable concentrations of CSF serotonin, adrenaline and metanephrine have yet to be determined.

## Conclusions

This study showed novel evidence of ventricular-lumbar CSF concentration gradients for dopamine, noradrenaline, 3-MT and NMN, and confirmed prior findings of gradients for DOPAC, HVA, and 5-HIAA concentrations. For these analytes, concentrations increased from the first to the last CSF fraction. These results contribute to the understanding of the neurobiology and can help to identify novel biomarkers in neuroscience. In future studies analysing CSF biogenic amines concentrations, standardisation of CSF collection and handling is essential for comparisons between studies.

## Supplementary Information

**Additional file 1: Fig. S1.** Concentrations of analytes displayed per patient in five consecutive CSF fractions. Every line presents the median concentration of that analyte for one patient in the five consecutive fractions of CSF. 1: 0-2 mL, 2: 2-4 mL, 3: 4-6 mL, 4: 6-8 mL, 5: 8-10mL. The blue line shows the overall median concentration of that analyte and the grey area the corresponding IQR. (A) L-DOPA (levodopa), (B) 3-OMD (3-o-methyldopa), (C) DA (dopamine), (D) DOPAC (3,4-Dihydroxyphenylacetic acid), (E) 3-MT (3-methoxytyramine), (F) HVA (homovanillic acid), (G) NA (noradrenalin), (H) NMN (normetanephrine), (I) MOPEG (3-Methoxy-4-hydroxyphenylglycol), (J) 5-HIAA (5-hydroxyindoleacetic acid). (K) albumin, (L) total protein, (M) glucose, (N) erythrocyte count and (O) leukocyte count.

**Additional file 2: Table S1.** Correlation of concentrations of analytes between plasma and CSF fractions. Spearman (rs) or Pearson (rp) correlation coefficient and significance for the relationship between plasma and CSF fractions. Correlation is significant at 0.05 level (*), 0.01 level (**) or 0.001 level (***)(2-tailed). L-DOPA: levodopa; 3-OMD: 3-o-methyldopa; DA: dopamine; DOPAC: 3,4-Dihydroxyphenylacetic acid; 3-MT: 3-methoxytyramine; HVA: homovanillic acid; NA: noradrenalin; NMN: normetanephrine; MOPEG: 3-Methoxy-4-hydroxyphenylglycol; 5-HIAA: 5-hydroxyindoleacetic acid; Qalb: ratio of CSF to plasma albumin concentration.

## Data Availability

The datasets used and analysed during the current study are available from the corresponding author on reasonable request.

## Abbreviations

3-MT: 3-Methoxytyramine
3-OMD: 3-o-methyldopa
5-HIAA: 5-Hydroxyindoleacetic acid
5-HT: Serotonin
Aβ: Amyloid-β
ABC: Anaesthetic Biobank of Cerebrospinal Fluid
ASA: American Society of Anaesthesiologists
BCB: Blood-CSF-barrier
BMI: Body Mass Index
CNS: Central nervous system
CSF: Cerebrospinal fluid
DA: Dopamine
DOPAC: 3,4-Dihydroxyphenylacetic acid
DTT: Dithiothreitol
HVA: Homovanillic acid
IgG: Immunoglobulin G
IQR: Inter quartile range
LC–MS: Liquid chromatography mass spectrometry
L-DOPA: Levodopa
LOQ: Limit of quantification
MN: Metanephrine
MOCA: Montreal Cognitive Assessment
MOPEG: 3-Methoxy-4-hydroxyphenylglycol
NA: Noradrenaline
NMN: Normetanephrine
PD: Parkinson’s disease
Qalb: CSF/blood albumin quotient
RCG: Rostrocaudal concentration gradient
