# Supplementary figures and images for: Concentration gradients of monoamines, their precursors and metabolites in serial lumbar cerebrospinal fluid of neurologically healthy patients determined with a novel LC–MS/MS technique
Source: Fluids Barriers CNS. 2023 Feb 13;20:13. doi: 10.1186/s12987-023-00413-8 (PMC9923930; doi:10.1186/s12987-023-00413-8)

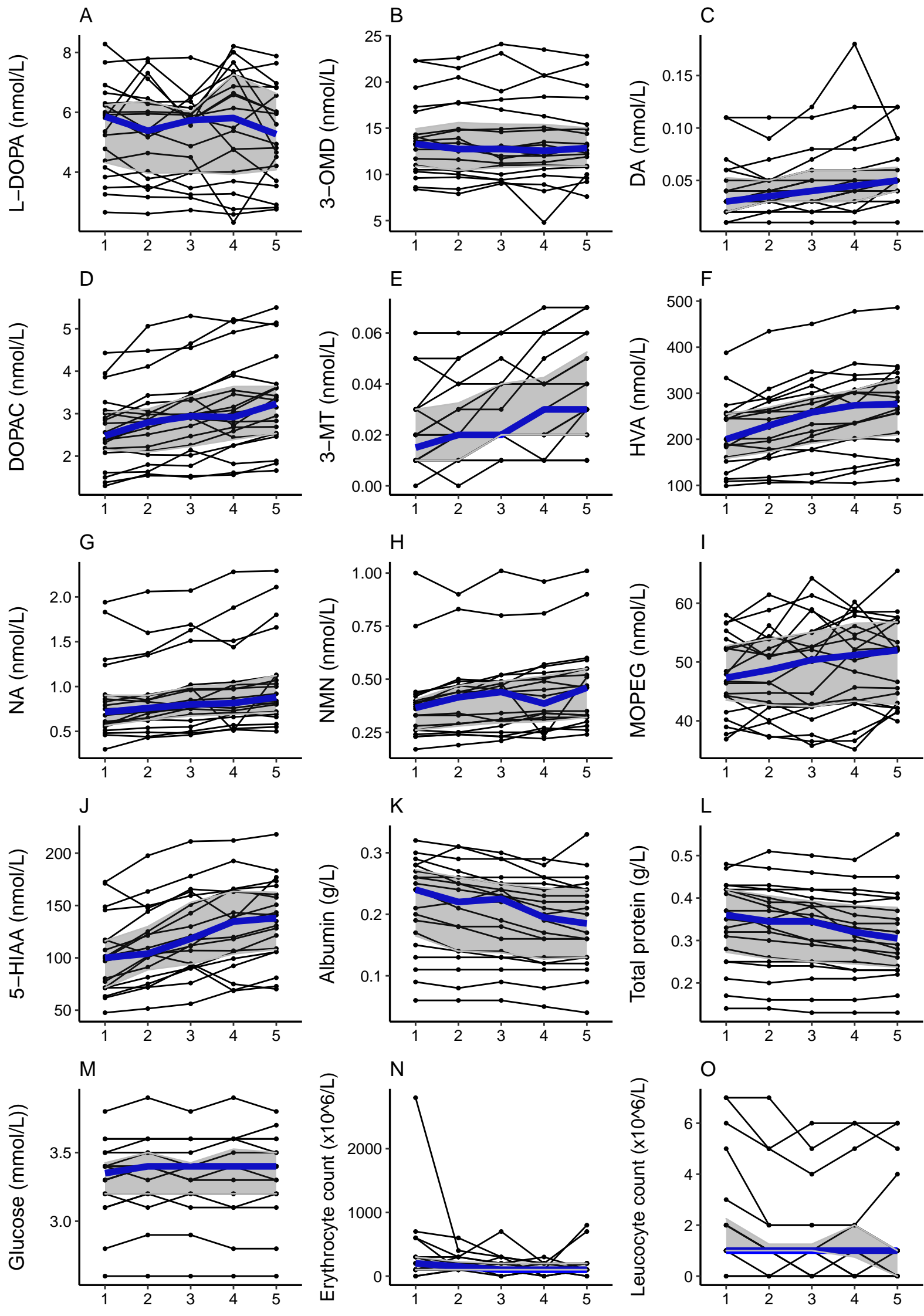

Supplement: Supplementary file 1 — Additional file 1: Fig. S1. Concentrations of analytes displayed per patient in five consecutive CSF fractions. Every line presents the median concentration of that analyte for one patient in the five consecutive fractions of CSF. 1: 0-2 mL, 2: 2-4 mL, 3: 4-6 mL, 4: 6-8 mL, 5: 8-10mL. The blue line shows the overall median concentration of that analyte and the grey area the corresponding IQR. (A) L-DOPA (levodopa), (B) 3-OMD (3-o-methyldopa), (C) DA (dopamine), (D) DOPAC (3,4-Dihydroxyphenylacetic acid), (E) 3-MT (3-methoxytyramine), (F) HVA (homovanillic acid), (G) NA (noradrenalin), (H) NMN (normetanephrine), (I) MOPEG (3-Methoxy-4-hydroxyphenylglycol), (J) 5-HIAA (5-hydroxyindoleacetic acid). (K) albumin, (L) total protein, (M) glucose, (N) erythrocyte count and (O) leukocyte count. [file 12987_2023_413_MOESM1_ESM.pdf]
