# Supplementary material for: Concentration gradients of monoamines, their precursors and metabolites in serial lumbar cerebrospinal fluid of neurologically healthy patients determined with a novel LC–MS/MS technique
Source: Fluids Barriers CNS. 2023 Feb 13;20:13. doi: 10.1186/s12987-023-00413-8 (PMC9923930; doi:10.1186/s12987-023-00413-8)
Supplement: Supplementary file 2 — Additional file 2: Table S1. Correlation of concentrations of analytes between plasma and CSF fractions. Spearman (rs) or Pearson (rp) correlation coefficient and significance for the relationship between plasma and CSF fractions. Correlation is significant at 0.05 level (*), 0.01 level (**) or 0.001 level (***)(2-tailed). L-DOPA: levodopa; 3-OMD: 3-o-methyldopa; DA: dopamine; DOPAC: 3,4-Dihydroxyphenylacetic acid; 3-MT: 3-methoxytyramine; HVA: homovanillic acid; NA: noradrenalin; NMN: normetanephrine; MOPEG: 3-Methoxy-4-hydroxyphenylglycol; 5-HIAA: 5-hydroxyindoleacetic acid; Qalb: ratio of CSF to plasma albumin concentration. [file 12987_2023_413_MOESM2_ESM.docx]

# **Supplemental Table 1** Correlation of concentrations of analytes between plasma and CSF fractions

| Plasma |  | CSF  0 - 2 mL | CSF  2 - 4 mL | CSF  4 - 6 mL | CSF  6 - 8 mL | CSF  8 - 10 mL |
| --- | --- | --- | --- | --- | --- | --- |
| Albumin | r_p_ | 0.168 | 0.128 | 0.148 | 0.065 | 0.068 |
|  | *p* | 0.479 | 0.592 | 0.533 | 0.785 | 0.776 |
| Total protein | r_p_ | 0.106 | 0.158 | 0.140 | 0.116 | 0.184 |
|  | *p* | 0.655 | 0.506 | 0.557 | 0.626 | 0.437 |
| Glucose | r_p_ | 0.609* | 0.635* | 0.638* | 0.633* | 0.645* |
|  | *p* | 0.004 | 0.003 | 0.002 | 0.003 | 0.002 |
| Leucocyte count | r_s_ | -0.302 | -0.275 | -0.308 | -0.278 | -0.135 |
|  | *p* | 0.196 | 0.240 | 0.187 | 0.235 | 0.571 |
| L-DOPA | r_p_ | -0.050 | -0.191 | -0.040 | -0.054 | -0.236 |
|  | *p* | 0.833 | 0.420 | 0.868 | 0.819 | 0.316 |
| 3-OMD | r_s_ | 0.866** | 0.869** | 0.891** | 0.876** | 0.884** |
|  | *p* | <0.001 | <0.001 | <0.001 | <0.001 | <0.001 |
| DA | r_s_ | -0.049 | -0.079 | -0.058 | 0.002 | -0.053 |
|  | *p* | 0.838 | 0.742 | 0.808 | 0.992 | 0.825 |
| DOPAC | r_p_ | 0.146 | 0.105 | 0.115 | 0.081 | 0.160 |
|  | *p* | 0.538 | 0.658 | 0.628 | 0.735 | 0.502 |
| 3-MT | r_s_ | 0.198 | 0.075 | 0.054 | 0.118 | 0.060 |
|  | *p* | 0.403 | 0.754 | 0.820 | 0.621 | 0.801 |
| HVA | r_p_ | 0.204 | 0.206 | 0.192 | 0.135 | 0.190 |
|  | *p* | 0.388 | 0.384 | 0.417 | 0.572 | 0.421 |
| NA | r_s_ | 0.144 | -0.132 | -0.135 | 0.032 | -0.111 |
|  | *p* | 0.543 | 0.578 | 0.569 | 0.895 | 0.640 |
| NMN | r_s_ | 0.095 | 0.046 | 0.108 | 0.138 | 0.009 |
|  | *p* | 0.691 | 0.848 | 0.652 | 0.561 | 0.970 |
| MOPEG | r_p_ | 0.420 | 0.550* | 0.443 | 0.435 | 0.417 |
|  | *p* | 0.065 | 0.012 | 0.051 | 0.055 | 0.068 |
| 5-HIAA | r_p_ | 0.187 | 0.085 | 0.093 | -0.072 | -0.011 |
|  | *p* | 0.431 | 0.722 | 0.696 | 0.763 | 0.962 |

# Spearman (rs) or Pearson (rp) correlation coefficient and significance for the relationship between plasma and CSF fractions. Correlation is significant at 0.05 level (*), 0.01 level (**) or 0.001 level (***)(2-tailed). L-DOPA: levodopa; 3-OMD: 3-o-methyldopa; DA: dopamine; DOPAC: 3,4-Dihydroxyphenylacetic acid; 3-MT: 3-methoxytyramine; HVA: homovanillic acid; NA: noradrenalin; NMN: normetanephrine; MOPEG: 3-Methoxy-4-hydroxyphenylglycol; 5-HIAA: 5-hydroxyindoleacetic acid; Qalb: ratio of CSF to plasma albumin concentration
